# Optimizing DCD Liver Grafts With Prolonged Warm Ischemic Time Using Stabilized Plasmin in a Static Cold Storage Orthotopic Rat Liver Transplant Model

**DOI:** 10.1097/TXD.0000000000001665

**Published:** 2024-07-05

**Authors:** Riley Kahan, Nader Abraham, Min Zhang, Valery Novokhatny, Isaac Alderete, Paul Cray, Fengming Chen, Qimeng Gao, Greta Cywinska, Ryan Neill, Kentaro Nakata, Ahmed Hassan, Caroline Rush, Jude Penaflor, Justin J. Pollara, Matthew G. Hartwig, Benjamin Hughes, Andrew S. Barbas

**Affiliations:** 1 Department of Surgery, Duke University, Durham, NC.; 2 Grifols Scientific Innovation Office, Grifols, NC.; 3 Department of Pathology, Duke University, Durham, NC.

## Abstract

**Background.:**

The clinical success of liver transplantation has led to increased demand, requiring further expansion of the donor pool. Therapeutic interventions to optimize organs from donation after circulatory death (DCD) have significant potential to mitigate the organ shortage. Dysfunction in DCD liver grafts is mediated by microvascular thrombosis during the warm ischemic period, and strategies that reduce this thrombotic burden may improve graft function. We hypothesized that the administration of the fibrinolytic enzyme plasmin to the donor organ during the cold storage period would reduce the thrombotic burden and improve DCD liver graft function.

**Methods.:**

In 2 separate cohorts, 32 syngeneic orthotopic rat liver transplants were performed in Lewis rats. Livers were procured from donors with 45 min of warm ischemic injury. Liver grafts were flushed with histidine-tryptophan-ketoglutarate preservation solution mixed with either plasmin (experimental group) or albumin (control group). All investigators were blinded to treatment group. After preparing the liver for implant using a modified cuff technique, the liver was stored for 1 h by static cold storage at 4 °C. Immediately before implantation, the liver graft was flushed, and this effluent was analyzed for fibrin degradation products to determine graft clot burden. Twenty-four hours following transplantation, animals were euthanized, and samples were collected.

**Results.:**

Recipient survival was significantly higher for DCD liver grafts treated with plasmin compared with control. Moreover, histology of liver graft tissue immediately before implant reflected significantly reduced congestion in plasmin-treated livers (score, mean ± SD: 0.73 ± 0.59 versus 1.12 ± 0.48; *P* = 0.0456). The concentration of fibrin degradation products in the final flush before implantation was significantly reduced in plasmin-treated livers (743 ± 136 versus 10 919 ± 4642 pg/mL; *P* = 0.0001), reflecting decreased clot burden in the graft.

**Conclusions.:**

The present study demonstrates that plasmin improves survival and may reduce thrombotic burden in DCD liver grafts with prolonged warm ischemic injury, meriting further study.

Liver transplantation is currently the only treatment option for patients with end-stage liver disease, and patient outcomes following transplantation have continued to improve over time. Because of the clinical success of liver transplantation, there is a growing discrepancy between the number of patients requiring transplantation and the number of available donor organs. To address this disparity, ongoing efforts have been directed toward expanding the donor pool including by increased utilization of high-risk donor livers, such as those from donation after circulatory death (DCD). Although dysfunction in DCD liver grafts is likely multifactorial, a key driver of the pathophysiology is thought to be the development of microthrombi in the terminal vasculature because of prolonged stasis of blood flow.^1^ This process is associated with early allograft dysfunction, the development of ischemic cholangiopathy, and graft failure requiring retransplantation.^2-4^ Thus, there is a need to develop therapeutic strategies to reduce the thrombotic burden in DCD liver grafts.

Preclinical work in this area has focused on the tissue plasminogen activator (tPA)-plasminogen axis.^5^ In vivo, this process describes the cleavage of the inactive zymogen plasminogen into plasmin by tPA. The catalytically active plasmin can then break up cross-linked fibrin into fibrin degradation products (FDPs). Most studies in the context of DCD livers have focused on the administration of tPA, which requires an additional source of plasminogen to be effective, usually in the form of fresh frozen plasma.^6^ An alternative approach, however, is to directly administer stabilized plasmin, which can dissolve persistent blood clots in the absence of plasminogen, simplifying administration and eliminating the need for potentially scarce blood products.^7,8^ Indeed, observations in lung showed that plasmin administration during ex vivo perfusion resulted in enhanced postperfusion physiological parameters.^9^

In this study, we hypothesized that the administration of plasmin would reduce the thrombotic burden in DCD liver grafts and improve function posttransplant. We tested this hypothesis using a model of prolonged warm ischemia (45 min) in a rat orthotopic liver transplant model. We demonstrate that plasmin administration is associated with a significantly improved survival and appears to reduce the thrombotic burden in liver grafts.

## MATERIALS AND METHODS

### Animals

Male Lewis rats (Charles River Laboratory, Wilmington, MA) weighing between 200 and 300 g were used in this study. All protocols were approved by the Duke University Institutional Animal Care and Use Committee. All animals were maintained in the Duke Laboratory for Animal Research in standard pathogen-free conditions and in accordance with the “Guide for the Care and Use of Laboratory Animals” published by the National Institutes of Health^10^ and with a standard diet of rodent chow and water supplied ad libitum.

### Experimental Procedure

#### Cohort 1

In cohort 1, after midline incision and open laparotomy were performed, donor animals were injected with heparin (300 U/kg) intravascularly. After 3–5 min, the portal vein was clamped with a microsurgery vascular clamp and the hepatic artery was ligated with 7-0 ties and divided to induce warm ischemic time. After 30 min, the donor liver was procured as previously described^11^ and prepared for implant via a modified vascular cuff technique.^12^ Preparation of the liver graft was performed at ambient temperature, rather than on ice, to account for the last 15 min of warm ischemia time. Immediately following the 45-min warm ischemic period, the liver was flushed with 200 mL of histidine-tryptophan-ketoglutarate (HTK) containing either plasmin or albumin through the portal vein. The graft was then stored on ice at 4 °C for 1 h. After 1 h, the graft was flushed again with 100 mL of pure HTK solution. The liver was then transplanted into the recipient using the modified vascular cuff technique as we have previously described.^11^ The bile duct anastomosis was performed using a stent between the recipient and donor bile ducts. No hepatic artery anastomosis was performed. Recipients were monitored and then euthanized on postoperative day 1 (POD1). A total of 12 transplants were conducted following the protocol for cohort 1.

#### Cohort 2

To replicate clinical DCD liver recovery more closely, where the initial warm ischemic period occurs completely in situ, cohort 2 was designed. As in cohort 1, a midline incision and open laparotomy were performed, followed by intravascular heparin injection (300 U/kg). Three to five minutes after heparinization, the portal vein was clamped with a microsurgery vascular clamp and the hepatic artery was ligated with 7-0 ties and divided to induce 45 min of warm ischemic time in situ. Following the 45-min warm ischemic period, the liver was flushed with 50 mL of HTK containing either plasmin or albumin through the portal vein. Then, the donor liver procurement was done as previously described,^11^ and the organ was prepared for implant via a modified vascular cuff technique^12^ while on ice. After cuffing, the graft was flushed for a second time with 50 mL of HTK containing either plasmin or albumin and then stored on ice at 4 °C for 1 h. After 1 h, the graft was flushed for a third and final time with 50 mL of HTK containing either plasmin or albumin. The effluent of the final flush was collected for measurement of FDPs. The liver and bile duct were biopsied at this point. The liver was then put back on ice while the recipient was prepared for implant (15 min), and the liver was then transplanted into the recipient using the modified vascular cuff technique as described. The bile duct anastomosis was performed by using a stent between the recipient and donor bile ducts. No hepatic artery anastomosis was performed. Recipients were monitored and then euthanized at POD1. A total of 20 transplants were done following the protocol for cohort 2.

Predetermined exclusion criteria included the following: (1) static cold storage period that exceeded 1.5 h, (2) warm ischemic period that exceeded 45 min, (3) major vascular injury with exsanguination intraoperatively, (4) major hepatic parenchymal injury, and (5) a significant anhepatic period (ie, time elapsed between excision of recipient liver and reperfusion of implanted liver that exceeded 20 min).

### Plasmin Administration

A 2 mL vial of 10 mg/mL plasmin suspended in a low-pH stabilizing solution (Grifols USA, LLC) or 10 mg/mL albumin control (Recombumin Elite; Albumedix Ltd, Nottingham, United Kingdom) was mixed into 200 mL of HTK solution (Custodiol; Essential Pharmaceuticals, LLC, Durham, NC) to make the preservation solution. The enzymatically inert albumin was used as a control to account for any possible effects of adding protein to the HTK solution, and similarly diluted by adding 2 mL (10 mg/mL) to 200 mL of HTK solution. In cohort 1, the 200 mL preservation solution ± plasmin/albumin was administered in a single flush immediately before 1 h of cold storage. In cohort 2, the preservation solution ± plasmin/albumin was aliquoted into 50 mL aliquots and 3 separate flushes of the liver were performed at the following time points: (1) at the end the 45-min warm ischemic period in situ, (2) after cuff placement, and (3) immediately preimplant. For experiments in both cohorts, both surgeon and investigator were blinded to treatment.

### Survival

The primary study outcome was recipient survival to 24 h. Rats were monitored continuously in the laboratory for the first 4 h following transplantation, and early deaths during this period were recorded. Survivors past this time point were transported back to the animal facility and assessed the following day.

### Liver Function

Blood was collected only from animals that survived until POD1 and was collected at the experimental endpoint. Serum alanine aminotransferase and aspartate aminotransferase were measured with a liver function panel using the Piccolo Xpress chemistry analyzer (Abaxis, Union City, CA). Lactate level was measured using the lactate plus handheld (Nova Biomedical, Waltham, MA) and blood glucose level was measured using the One touch Verio flex handheld glucometer (Lifescan IP holdings, LLC, Malvern, PA).

### Fibrin Degradation Product

For cohort 2 animals, FDP concentration was assessed using the Rat FDP ELISA Kit (Abbexa Ltd, Cambridge, United Kingdom) according to the manufacturer’s instructions. FDP concentration of the effluent of the final flush of the liver before implant was measured to detect presence or absence of microthrombi in the grafts. Effluent was assayed in triplicate. The optical density was calculated against a standard curve and interpolated to determine concentration of FDP in each effluent sample.

### Histopathology

Liver graft tissue was collected preimplant from cohort 2 animals; liver tissue was only collected posttransplant if the animal survived to the POD1 endpoint. Tissue for histology was immediately fixed in 10% neutral buffered formalin and embedded in paraffin blocks. Each sample was then sectioned and stained with hematoxylin and eosin to evaluate morphological changes and fibrin deposition in the tissue (Histoserv, Inc., Germantown, MD). Tissue slides were graded for congestion, vacuolization, and necrosis by a blinded liver pathologist using the Suzuki Liver Injury Scoring System.^13^ Liver tissue was also scored for fibrin deposition as previously described.^14^

### Statistical Analysis

Data are represented by mean ± SD or box plots with whiskers depicting minimum to maximum. The Mantel-Cox log-rank test was used to examine the survival between the control and experimental groups. Comparison between groups for other parameters were performed using unpaired Student’s *t* test or Mann-Whitney *U* test with Prism 9, GraphPad Software (San Diego, CA). Significance was defined as *P* values of less than 0.05. The FDP analysis is derived only from cohort 2 because this liver flush effluent was not collected in cohort 1. For all other analyses, data were analyzed from cohorts 1 and 2 in aggregate.

## RESULTS

### Liver Graft Injury and Function

**FIGURE 1.**
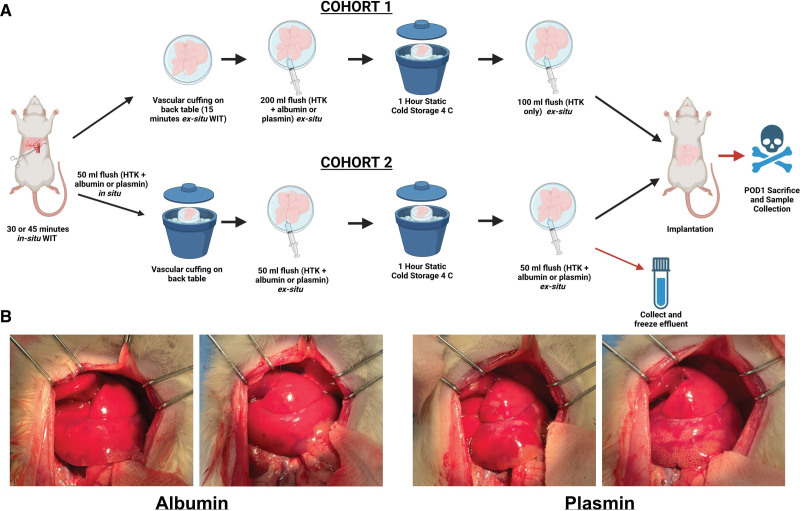
Experimental design for albumin/plasmin administration. A, Cartoon depicts the 2 administration protocols used in this study. A total of 17 albumin-treated and 15 plasmin-treated livers were transplanted. B, Representative gross images of transplanted livers at time of reperfusion for albumin- and plasmin-administered groups. No obvious differences were observed in graft homogeneity upon reperfusion. HTK, histidine-tryptophan-ketoglutarate; POD1, postoperative day 1; WIT, warm ischemic time.

**FIGURE 2.**
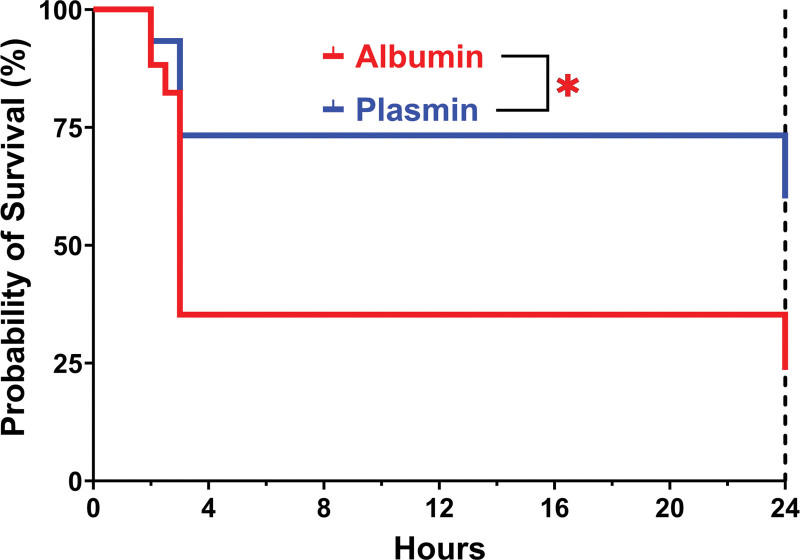
Participant survival following orthotopic liver transplant. Analysis of survival probability to the postoperative day 1 (POD1) endpoint revealed higher rate of survival for plasmin-administered participants compared with albumin-administered controls (*P* = 0.0316). Two participants in each group were found dead at 24-h posttransplant.

Gross morphology and tissue color appeared similar among both groups after reperfusion, and no apparent difference between cohorts was noted (Figure 1). Posttransplant, most recipients exhibited one of 2 phenotypes: the animal either decompensated with lethargy and slowed breathing within 2–4 h posttransplant, or the animal displayed relatively healthy clinical signs until POD1 euthanize. Animals that received plasmin had a significantly higher survival to the POD1 endpoint when compared with control animals (Mantel-Cox log-rank test; *X*^2^ [1, N = 32] = 4.622; *P* = 0.0316) (Figure 2). Median survival time for albumin control participants was approximately 3 h posttransplant, in contrast to the >24 h median survival observed for plasmin-administered participants. We note that 2 participants in each group were discovered dead at 24 h posttransplant, expiring at an unknown time during the interval between observations at 4 and 24 h. Limited necropsies were conducted on participants that died within 4 h posttransplant, finding no conclusive cause of death. Despite the frequent presence of blood in the abdomen, in all cases anastomoses were observed intact.

**FIGURE 3.**
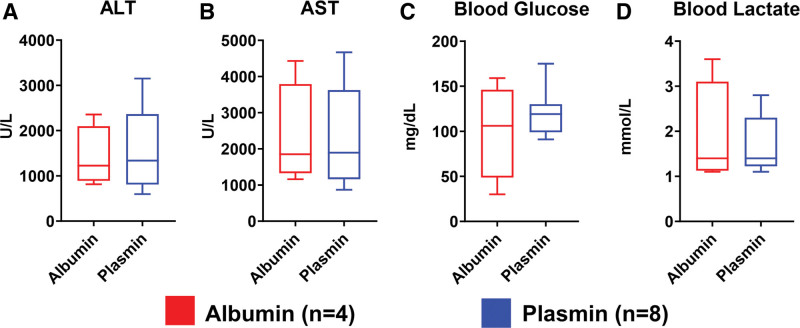
Posttransplant measures of liver function. A and B, ALT and AST displayed comparable posttransplant levels between both control and plasmin-administered groups. C, Blood glucose levels were equivalent between albumin and plasmin groups. D, Do apparent difference between groups was observed in mean blood lactate levels. ALT, alanine aminotransferase; AST, aspartate aminotransferase.

Liver function tests of participants that survived to POD1 revealed no significant differences in transaminases between treatment groups. Specifically, mean aspartate aminotransferase was 2323 ± 1442 U/L for albumin controls versus 2307 ± 1366 U/L for plasmin-treated participants (Figure 3A; *t* test; *t* = 0.018, *df* = 10; *P* = 0.986). Similarly, mean alanine aminotransferase was 1405 ± 667.3 U/L for albumin controls versus 1569 ± 899.5 U/L for plasmin-treated participants (Figure 3B; *t* test; *t* = 0.32, *df* = 10; *P* = 0.756). A minor, although nonsignificant, increase in blood glucose level was observed in plasmin-treated participants (121 ± 26 mg/dL) relative to albumin-administered participants (100.3 ± 53 mg/dL) (Figure 3C; *t* test; *t* = 0.931, *df* = 10; *P* = 0.374). Lactate levels were similarly equivalent between control and plasmin-treated participants, with mean levels of 1.875 ± 1.17 and 1.675 ± 0.63, respectively (Figure 3D; *t* test; *t* = 0.393, *df* = 10; *P* = 0.703). Lastly, no difference was observed in the volume of abdominal ascites in a subset of albumin- and plasmin-administered participants at the POD1 endpoint (data not shown; *t* test; *t* = 0.0267, *df* = 6; *P* = 0.98).

**FIGURE 4.**
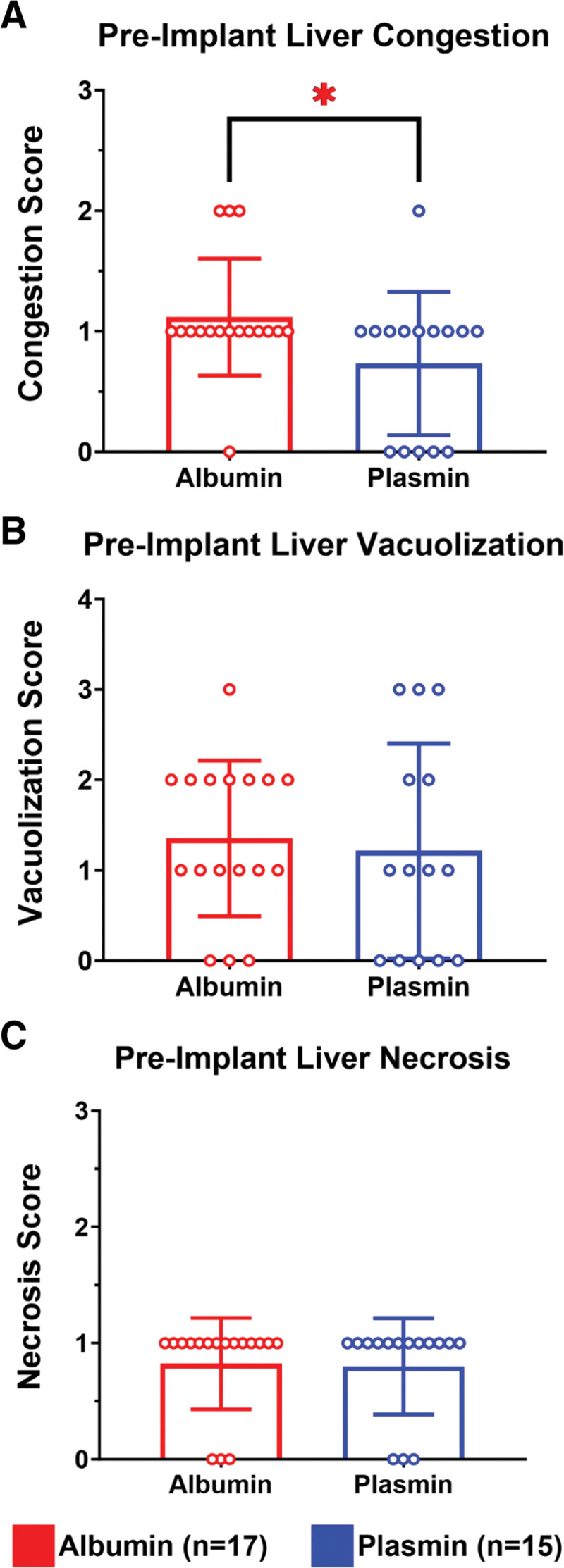
Preimplant liver histology. A, Congestion scores in plasmin-administered livers exhibited a modest, although significant, reduction pretransplant (*P* = 0.0456). B and C, Preimplant vacuolization and necrosis scores were otherwise equivalent between treatment groups.

**FIGURE 5.**
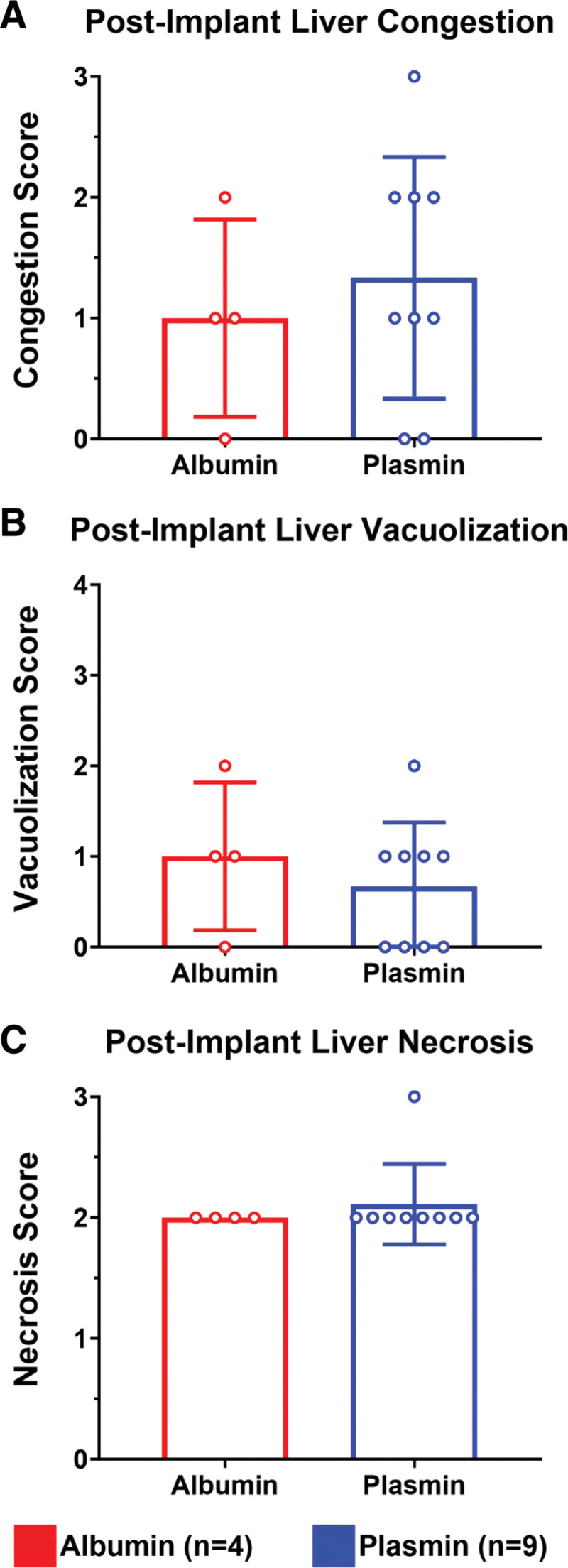
Postimplant liver histology. A, Quantification of liver congestion postimplant revealed no apparent different between control and plasmin-administered groups. B, Similarly, no difference in vacuolization was observed between groups. C, Although necrosis scores were elevated posttransplant, no difference was observed between groups.

**FIGURE 6.**
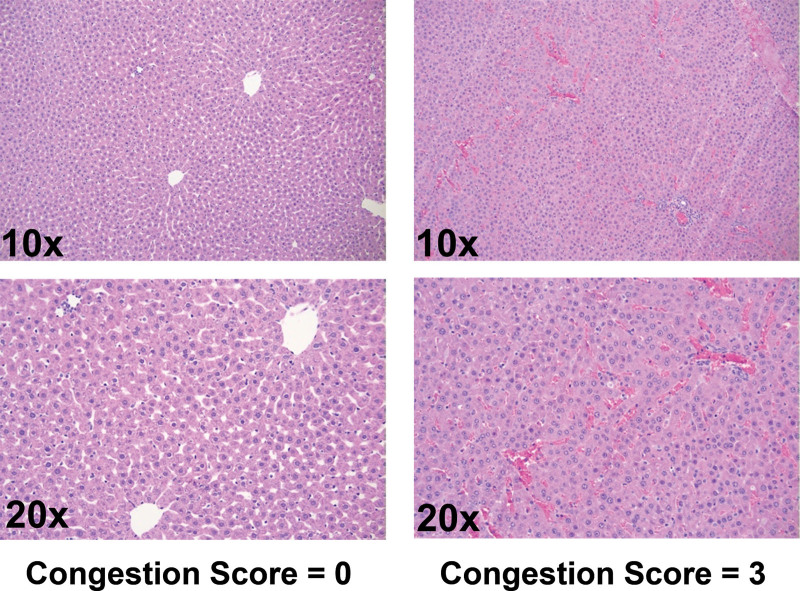
Representative liver histology examples. Representative images show differential severity of liver congestion corresponding to different Suzuki scores used for histological evaluation.

Histological grading was conducted on tissue biopsies collected before and after liver transplantation. Analysis of tissue revealed significantly reduced vascular congestion in plasmin-treated participants before implantation, with a mean congestion score of 0.73 ± 0.59 versus 1.12 ± 0.48 in albumin-treated control participants (Figure 4A; Mann-Whitney *U* test; *P* = 0.0456). Tissue samples, although, displayed similar levels of necrosis and vacuolization between groups (Figure 4B and C). Posttransplantation, participants that survived to POD1 demonstrated comparable congestion scores between groups (Figure 5A; Mann-Whitney *U* test; *P* = 0.72). Comparison of postimplant vacuolization and necrosis scores similarly revealed no apparent differences between groups (Figure 5B and C; Mann-Whitney *U* test; *P* = 0.678 and *P* > 0.999, respectively). Representative images in Figure 6 show differential congestion by score.

### Plasmin Efficacy

**FIGURE 7.**
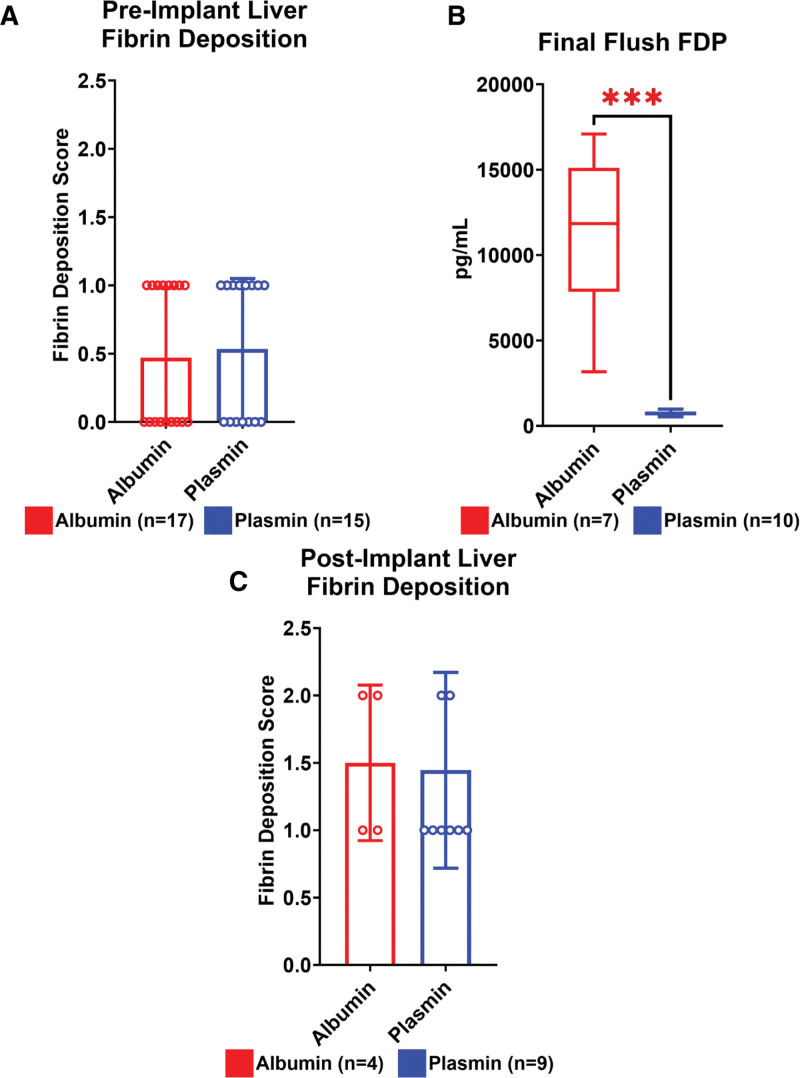
Plasmin activity and fibrin/FDP abundance. A, Histological evaluation of fibrin deposition before graft implant revealed no apparent difference between groups. B, FDP concentration measured in the effluent of the final liver flush before implant revealed dramatically reduced concentration of FDP (cohort 2 only). C, Posteuthanized histological scoring of fibrin deposition from postoperative day 1 (POD1) survivors revealed moderately elevated levels of deposition compared with preimplantation that did not differ between groups. FDPs, fibrin degradation products.

The presence of fibrin was quantified histologically and via ELISA pretransplantation and posttransplantation. Preimplant fibrin deposition scores were comparable between albumin- and plasmin-administered groups (Figure 7A; Mann-Whitney *U* test; *P* > 0.999). The concentration of FDPs in the effluent of the final graft flush immediately before implant was quantified by ELISA. Significantly reduced FDP was observed in plasmin-treated participants versus albumin-treated controls, finding mean FDP concentrations of 743 ± 136 versus 10 919 ± 4642 pg/mL, respectively (Figure 7B; Mann-Whitney *U* test; *P* = 0.0001). Simple linear regression was additionally performed to evaluate whether FDP concentration in effluent correlated with posttransplant survival time only in albumin-treated participants, finding no apparent correlation (data not shown; *r*^2^ = 0.155). Finally, histological examination of control and plasmin-treated grafts demonstrated comparable posttransplant fibrin deposition (Figure 7C; Mann-Whitney *U* test; *P* = 0.922).

## DISCUSSION

In this study, we demonstrate an apparent survival benefit associated with plasmin administration to DCD liver grafts with a significant warm ischemic injury of 45 min. To our knowledge, survival with this magnitude of ischemic injury in a rat liver transplant model has only been previously achieved with the use of normothermic machine perfusion.^15^ Interestingly, plasmin administration appears to reduce the clot burden in DCD grafts, reflected by a more than a 10-fold decrease in FDP concentration in plasmin-treated grafts (Figure 7). Although increased FDP might instead be expected with plasmin administration, the marked reduction in FDP we report could be attributable to progressive proteolytic cleavage of FDP by plasmin into undetectably small fragments. Indeed, such time-dependent cumulative degradation has been previously reported among the first mechanistic descriptions of plasmin activity.^16,17^ Although others report that plasmin administration elicits increased FDP, we note that samples were collected 10 min postadministration in that study.^9^ It is additionally possible that, as our samples were collected following multiple organ flushes, much of the microvascular clot burden had already been mobilized and cleared from the liver, revealed as lower FDP levels in the plasmin group. Regardless, although the present study did not resolve any apparent relationship, it would be of considerable interest to determine whether preimplantation FDP levels correlate with posttransplant outcomes, and merits additional investigation.

In addition, future investigation could explicitly determine whether ex vivo administration represents an ideal platform for the therapeutic actions of plasmin, particularly given the absence of the enzyme α2-antiplasmin that negatively regulates plasmin activity.^17^ To this point, histological evaluation of fibrin in posttransplant livers at POD1 revealed similar levels of deposition, despite the marked reduction of FDP observed in the final organ flush. We attribute this elevated postprocedure deposition score to residual circulating fibrin in vivo, perhaps secondary to the implantation procedure, that reflects the tight regulatory control implicit to the tPA-plasminogen axis. Indeed, this regulatory control could be leveraged to permit additional postsurgical plasmin administration to further mitigate clot burden in the graft. Although doing so would require careful titration, the presence of circulating inhibitory enzymes could act to restrict the manifestation of uncontrolled bleeding while further abrogating microvascular clot burden in the graft.

With the establishment of this model, we introduce a clinically relevant approach to study novel therapeutics to improve outcomes in liver transplantation using high-risk DCD liver grafts. This model will allow further investigation into novel treatment options that can lower the risk of primary nonfunction and early allograft dysfunction inherent to transplanting DCD livers.^18,19^ Our findings also demonstrate an association between reduced clot burden in the graft and improved posttransplant outcome and are consistent with recent findings of Watson et al.^20^ In their study, livers with lower levels of d-dimer present in the perfusate during normothermic machine perfusion, reflective of a lower occult fibrin burden, demonstrated better clinical outcomes following transplantation.^20^ It is unclear from our data specifically how plasmin treatment promoted participant survival, although the likeliest explanations are either attenuation of ischemia-reperfusion injury, reduced early allograft dysfunction, or both. Future studies could therefore conduct additional periodic liver function tests posttransplant to determine the extent to which increased survival is the product of reduced early allograft dysfunction. As well, further study could quantify biochemical markers of ischemia-reperfusion injury including complement activation and caspase expression. In terms of safety, a theoretical concern with plasmin administration is a higher risk of bleeding complications. In our study, we did not observe an increase in postoperative internal bleeding when compared with controls, suggesting that plasmin is likely safe to use to treat donor grafts in the context of liver transplantation, particularly when administered to the liver ex vivo. This supports previous findings that plasmin not only exhibits more effective thrombolytic activity than plasminogen activators like tPA but that plasmin can also achieve thrombolysis without attendant coagulopathy or other bleeding complications.^6^

This study has several limitations that warrant discussion. First, because of the nature of the study as a pilot, animals in cohorts 1 and 2 were treated with slightly different protocols with respect to how warm ischemia was applied and how plasmin was administered. Despite this, the survival of control groups for each cohort was similar, suggesting an overall similar magnitude of graft injury. Second, because of study design, we were not able to determine the degree of liver dysfunction in animals that died before the study endpoint. As such, a survival bias was present for outcomes measured at POD1, and thus no differences were obvious in liver function tests or histology between control and treatment animals who survived to the endpoint. Future studies should incorporate an early time point (such as 2-h postreperfusion) to help clarify this matter, as well as additional monitoring throughout the 24-h postoperative period.

In conclusion, we present evidence that stabilized plasmin appears to have a beneficial effect in a rat DCD liver transplant model with prolonged ischemic injury. Although further studies are needed to more firmly establish the therapeutic value of plasmin, particularly in clinically relevant large animal models, we present compelling evidence for the use of plasmin to improve static cold storage preservation solutions in the context of DCD organ recovery.
